# The Preparation, Microstructure, and Wet Wear Properties of an Fe55-Based Welding Layer with the Co-Addition of 0.01 wt% CeO_2_ and 1.5 wt% SiC Particles Using the Plasma Beam Spraying Method

**DOI:** 10.3390/ma16237439

**Published:** 2023-11-29

**Authors:** Liang Yu, Fuming He, Xinbin Liu, Yanli Jiang, Mengmeng Sui, Xiuling Cao, Zhengbing Meng

**Affiliations:** 1 Key Laboratory of New Processing Technology for Nonferrous Metals & Materials, Guilin University of Technology, Guilin 541004, China; 2010054@glut.edu.cn (L.Y.); 18577362994@163.com (F.H.); 2010043@glut.edu.cn (Y.J.); suimengmeng0808@163.com (M.S.); 2 Collaborative Innovation Center for Exploration of Nonferrous Metal Deposits and Efficient Utilization of Resources, Guilin University of Technology, Guilin 541004, China; 3 Guangxi Modern Industry College of Innovative Development in Nonferrous Metal Material, Guilin University of Technology, Guilin 541004, China; 4 School of Intelligent Manufacturing and Mechanical Engineering, Hunan Institute of Technology, Hengyang 421002, China; liuxinbin@hnit.edu.cn; 5 Hebei Technology Innovation Center for Intelligent Development and Control of Underground Built Environment, Shijiazhuang 050031, China; 6 School of Exploration Technology and Engineering, Hebei GEO University, Shijiazhuang 050031, China

**Keywords:** plasma beam spraying method, co-addition, CeO_2_, microstructure, wear performance

## Abstract

Severe erosion wear is found on valve spools, which threatens the safety and reliability of these units. The use of the plasma beam spraying surfacing method can significantly improve the corrosion resistance and sealing performance of hydraulic valve spools, reduce material waste, and reduce maintenance costs. The effects of the co-addition of CeO_2_ and SiC particles on the morphology, surface cracks, microstructure, precipitated phases, and wear property of plasma-beam-sprayed Fe55-based coatings on 1025 steel were investigated using OM, EDS, ultra-deep field microscopy, and a wet sand rubber wheel friction tester, respectively. The dendrite exhibited a directional growth pattern perpendicular to the substrate and the transitional states of the microstructure with the co-addition of CeO_2_ and SiC particles. CeO_2_ or SiC reduced the liquid phase diffusion coefficient D_L_ of Cr and C and resulted in a decrease in the G/R ratio. The dendrites changed into equiaxed grains. The main phase composition of the Fe55 welding layer was Cr_7_C_3_, γ-Fe. The martensite in the surfacing layer and the carbides formed Cr_7_C_3_, which can improve the hardness of the surfacing layer. The grain boundaries consisted mainly of a reticular eutectic structure. The uniform distribution of the Cr_7_C_3_ hard phase in the Fe55+1.5 wt% SiC+0.01 wt% CeO_2_ resulted in a uniformly worn surface. The sub-wear mechanisms during the friction process were micro-ploughing and micro-cutting. The hardness and toughness of Fe55+1.5 wt% SiC+0.01 wt% CeO_2_ were well-matched, avoiding excessive micro-cutting and microplastic deformation. A low content of CeO_2_ could lead to the formation of equiaxed grain and effectively improve the uniformity of the microstructure. The wear-resistant layer of Fe55+1.5 wt% SiC+0.01 wt% CeO_2_ can effectively improve the service life and long-term sealing performance of the valve spools.

## 1. Introduction

The micro-cutting of mineral particles during slurry transportation could cause gaps to form and lead to the line seal’s failure [1]. The failure of the sealing is caused by the combined effect of the abrasive particles in the two-phase flow of slurry. The main wear behaviors are scratching of the sealing surface, liquid corrosion, and erosion [2]. The manufacture and repair of the wear-resistant valve-sealing surface is of interest to researchers [3]. At present, the laser cladding method (LCM) and the plasma beam spraying method (PSM) are usually applied to manufacture and repair the carbide material layer at the valve sealing surface [4]. The LCM uses a laser to form a molten pool on the surface of the substrate and the metal powder is sprayed to achieve metallurgical bonding. The PSM forms a high temperature plasma arc on the surface of the substrate, and the metal powders are heated using a plasma arc to form a liquid droplet that is sprayed on the surface of the substrate to achieve metallurgical bonding. The metallurgical bonding effect of the PSM is better than that of the LCM. Therefore, the PSM is widely used for valve repair [5].

Fe-based powders are widely used in the field of surface repair and enhancement because of their low price and achievable melting temperature. Studies have reported the type of enhanced phase, content, and elemental composition of alloy powders. The PSM’s parameters have comprehensive effects on the microstructural properties of the welding layer [6]. Ning et al. reported on how the metal carbides produced through SiC decomposition, combined with the diffusion elements of the matrix, have a positive effect on the increase in hardness [7]. Not only might the addition of SiC be used in the enhanced phase, but also as sources of other carbides. A study by Zhang et al. showed that the formation of graphite in the free stage after the decomposition of SiC particles in steel at high temperature leads to the generation of defects and the degradation of the performance [8]. This suggests that the decomposition of SiC should be inhibited to avoid defects. Chen et al. showed that CeO_2_ reduces cracks and defects caused by Ti(C,N), in addition to the weld layer, and contributes to the improvement in the hardness and wear properties [9]. They provide evidence that CeO_2_ can inhibit defects after the addition of an enhanced phase. A study by Gao et al. on the mechanism by which CeO_2_ refines the grain size in Ni-based welding indicated that Ce hinders the growth of grains by generating a drag effect if it accumulates along grain boundaries. This process forms many nucleation sites to improve the microstructure. In addition, the addition of Ce may also reduce the fluidity of the molten pool, resulting in a weakening of the grain-refining effect [10]. However, the low fluidity might decrease the agglomeration rate of the particles in the molten steel and provide the possibility of adding ceramic particles without defects. Cai et al. co-added CeO_2_ and nano-TiC in the welding layer. The results show that 3 wt% CeO_2_ promoted the melting and decomposition of the nano-TiC micro surface and contributed to the uniform distribution of the enhanced phase. However, the excessive amount of CeO_2_ led to the decomposition of the enhanced phase, and the amount of CeO_2_ added should be carefully evaluated [11]. This indicates that the amount of co-added CeO_2_ and ceramic particles should match. Nevertheless, these researchers focused on the improvement in surface hardness and the reduction in the volume of wear. The uniform distribution of the enhanced phase, which can promote microstructural homogenization, has been proven. CeO_2_ can act as nucleation site for carbides and promote the refinement of M_7_C_3_ [12]. Therefore, the co-addition of CeO_2_ and ceramic particles will promote the homogeneity of hardness, which provides theoretical support for the preparation of valve-sealing surface materials with a uniform microstructure.

In the above studies, the researchers found that the addition of CeO_2_ promotes the uniform distribution of ceramic particles. These uniformly distributed ceramic particles result in the uniform nucleation of the grains and possibly uniform hardness. The failure mechanism of the sealing surface was investigated in depth by Chen et al. [3]. However, the wear’s uniformity has not received sufficient attention from other researchers. But uniform a microstructure was observed their works [7,8,9,10,11,12]. Li et al. indicated that the size and speed of particles are positively related to the wear rate, and in slurry transportation, the size and speed of particles are constrained within a small range. One important result reported is that the soft target wear depth was greater than the hard target wear depth [13]. It provided a path to improve the performance of valve sealing and a uniform distribution of the hard phase is the key.

Although SiC had been reported to be used as a reinforcement, correspondingly, in welding layers prepared using LCM [7,14], the effect of the co-addition of SiC and CeO_2_ on the microstructure and wet wear of Fe-based welding layers has rarely been investigated. In this study, we focus on investigating the influence of microstructures by SiC and CeO_2_. We prepared three different welding layers with different content additions of CeO_2_ and SiC particles using PSM in Section 3.2 of this article and compared the microstructure of three different welding layers. In Section 3.3, we compared the wear performance of three different welding layers using a wet sand rubber wheel friction tester. In Section 3.4, the effect of the co-addition of CeO_2_ and SiC on the wear mechanism was investigated. Based on these studies, it would be possible to prepare a layer with a uniform microstructure through the co-addition of CeO_2_ and SiC particles and to achieve a material with uniform wear.

## 2. Materials and Methods

**Figure 1:**
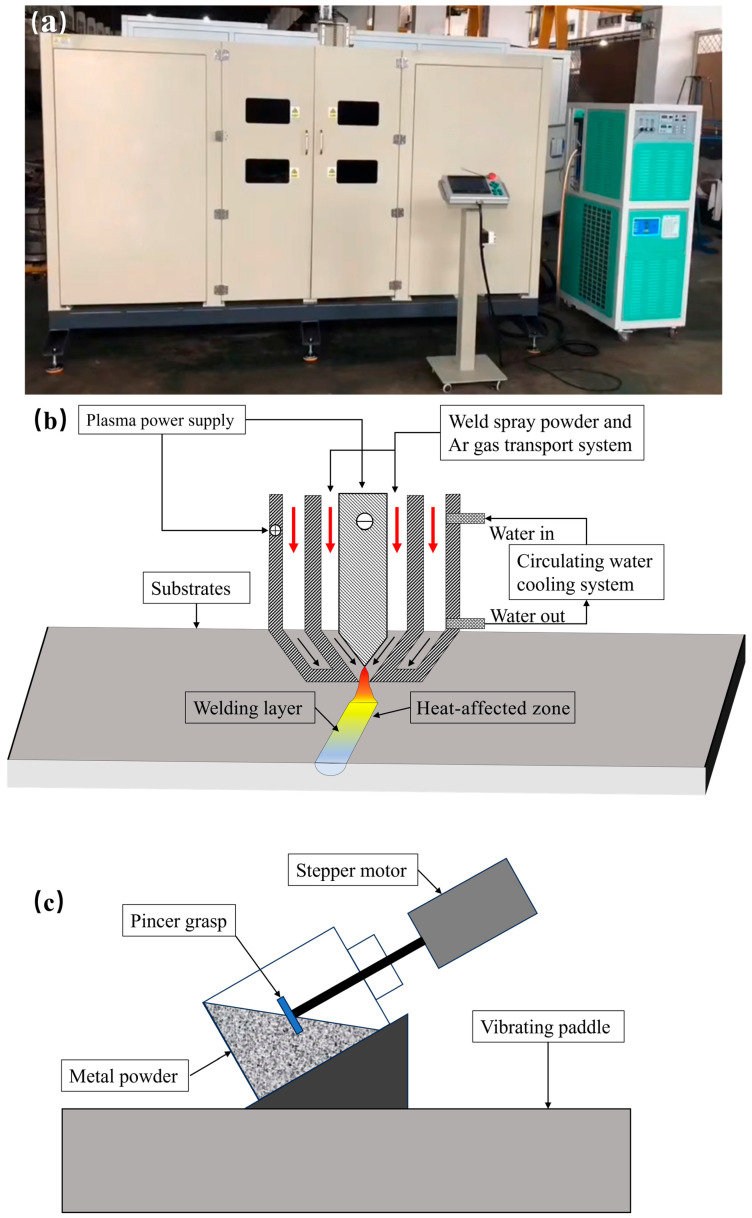
Schematic diagram of 3D PSM system and process. (**a**) 3D PSM system; (**b**) schematic diagram of the 3D PSM process; (**c**) vibrating paddle stirrer.

Annealed 1025 carbon steel is commonly used as the base material for valve spools. In this paper, a 1025 steel plate of size 100 mm × 50 mm × 10 mm was selected as the substrate. The surface of the substrate was ground to remove the oxide layer before the surface was sprayed. The Fe55 alloy powder had suitable hardness and high Ni content to avoid corrosion, and widely used as wear-resistant layer. Therefore, the Fe55 (purity > 99%, *d*_50_ = 150~200 μm, Hebei Hangba Metal Materials Co., Ltd., Xingtai, China) was considered as the matrix material of the welding layer. CeO_2_ (purity > 99%, *d*_50_ = 100 nm, Hebei Hangba Metal Materials Co., Ltd., Xingtai, China) was used to modify the microstructure. SiC (purity > 99%, *d*_50_ = 100~150 μm, Hebei Hangba Metal Materials Co., Ltd., Xingtai, China) was used as the enhanced phase. Table 1 and Table 2 show the chemical composition of raw materials. Firstly, with the low addition of CeO_2_, the powders were premixed according to the following steps to achieve a homogeneous mixing of CeO_2_ with the alloy powders. To avoid the brittle fracture of SiC particles during the ball milling stage, the ball milling speed and time in the premixing stage were set at a low value. Amounts of 2 g of Fe55 alloy powder, 1.5 g of SiC, and 0.01 g of CeO_2_ powder were mixed in an Al_2_O_3_ ball mill jar for 30 min at 250 rpm. The mixed powder was mixed with the remaining Fe55 alloy powder in a vibrating paddle stirrer (self-made) with a speed of 3000 rpm for 1 h and a vibration frequency of 15 Hz, as shown in Figure 1c.

**Table 1:** Chemical composition of Fe55.

Elements	C	Si	B	Cr	Ni	Fe
wt%	0.7–1.0	3.0–4.0	3.5–4.0	16–18	10–13	Balance

**Table 2:** Chemical composition of powders.

Samples	Fe55 wt%	SiC wt%	CeO_2_ wt%
Fe55	100	0	0
Fe55 + 1.5 wt% SiC	98.5	1.5	0
Fe55 + 1.5 wt% SiC + 0.01 wt% CeO_2_	98.49	1.5	0.01

Figure 1 showed a schematic diagram of the 3D PSM system and process (DML-V03BD, Shanghai Duo Mu Industrial Co., Shanghai, China). Spraying was performed using a 3D PSM system. The steps were as follows: (1) the homogeneous powder mixtures (Fe55, Fe55+1.5 wt% SiC, Fe55+1.5 wt% SiC+0.01 wt% CeO_2_) were loaded into the spray tank with continuous stirring using a stirring paddle; (2) to prevent thermal stress cracks in the welding layer, the 1025 steel plate was preheated to 300 °C; and (3) the surface oxidation layer was cleaned again before the spraying process. Before the spraying process, the welding parameters were determined via a pre-experiment. An Ar atmosphere was used to avoid the oxidation of the welding surface with a gas flow rate of 5 L/min. The higher flow rate might lead to intense stirring and resulted in the molten steel experiencing splashing. The ion gas flow rate was 3.5 L/min to avoid the heat effect zone of substrate being too deep and to decrease the bonding between the substrate and welding layer. The welding current was 95 A, the scan rate was 6 mm/s to avoid coarse grain caused by high temperature gradient, and the powder transfer rate was 1.5 g/s. All parameters were monitored using the spraying system shown in Figure 1a.

The samples with dimensions of 10 mm × 10 mm × 8 mm were cut from the substrate through wire cutting. The Ni and Cr contents of Fe55 were high and resulted in high corrosion resistance. After being ground and polished, the samples were etched for 30 s using an alcoholic nitric acid etching solution with a concentration of 4 vol.%. The samples were cleaned with anhydrous alcohol and dried to observe the microstructure using an optical microscope (BMM-420, Shanghai Batuo Instrument Co., Ltd., Shanghai, China). The microstructure characterization of the samples was observed using a Zeiss GeminiSEM 300 field-emission scanning electron microscope (Oberkochen, Germany) equipped with an energy-dispersive X-ray spectrometer (Oberkochen, Germany). The ultra-deep field microscopy (μsurf explorer, NanoFocus AG, Oberhausen, Germany) was used to characterize the worn surface, according to a study by Chen et al. [9]. The polished samples were tested for micro-Vickers hardness using a semi-automatic hardness tester (AMH43, LECO, Laboratory Equipment Co., St. Joseph, MI, USA). The measurement spacing from substrate to coating was 200 μm. The load was 0.5 kgf and the holding time was 15 s. The indentation depth was determined by the 4-point spacing; each sample was tested 3 times and we calculated the average value.

**Figure 2:**
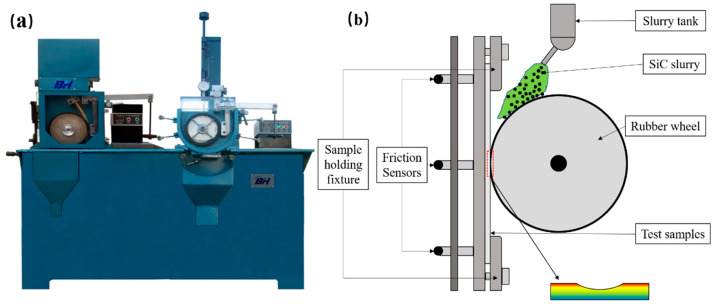
LGM-225 wet sand rubber wheel friction tester and schematic diagram of wear process (**a**) tester; (**b**) schematic diagram of wear process.

To test the wear resistance and investigate the wear mechanism, the wet sand rubber wheel friction tester shown in Figure 2 (LGM-225, Jinan Liangong Test Technology Co., Jinan, China) was used to test the wear performance of different samples. The samples were polished to a roughness of Ra < 0.5 μm before the wear test. The SiC particles (purity > 99%, *d*_50_ = 75 nm, Hebei Hangba Metal Materials Co., Ltd., Xingtai, China) were used as the abrasive. The wear load of 20 N was applied and the wear time was 10 min for 3 times. The rubber wheel linear speed was 3 m/s.

## 3. Results and Discussion

### 3.1. Morphology and Chemical Composition of the Raw Powders

**Figure 3:**
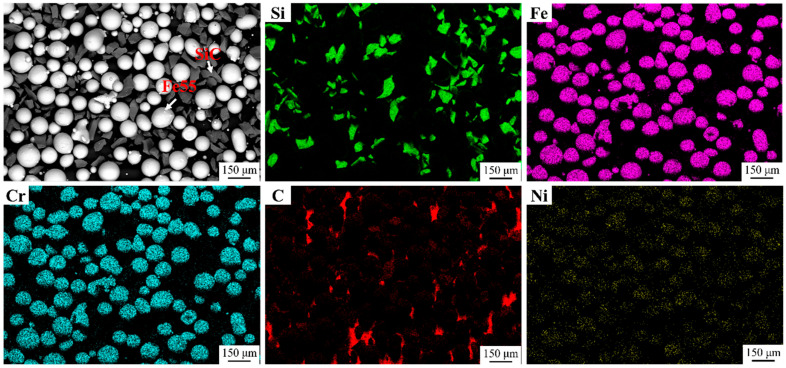
Powder microstructure and EDS mapping of Fe55+1.5 wt% SiC.

Figure 3 shows the SEM micrographs and EDS mapping of Fe55+1.5 wt% SiC powders. The sizes of Fe55 and SiC were about 150 μm. The ball milling speed was low. Most of the SiC had not broken during the ball milling process and remained prismatic. A small part of SiC had broken and smaller SiC with 40~50 μm were generated. The rich areas of Fe and Cr overlapped with Ni, indicating that Fe55 was mainly composed of Fe, Cr, and Ni elements.

**Figure 4:**
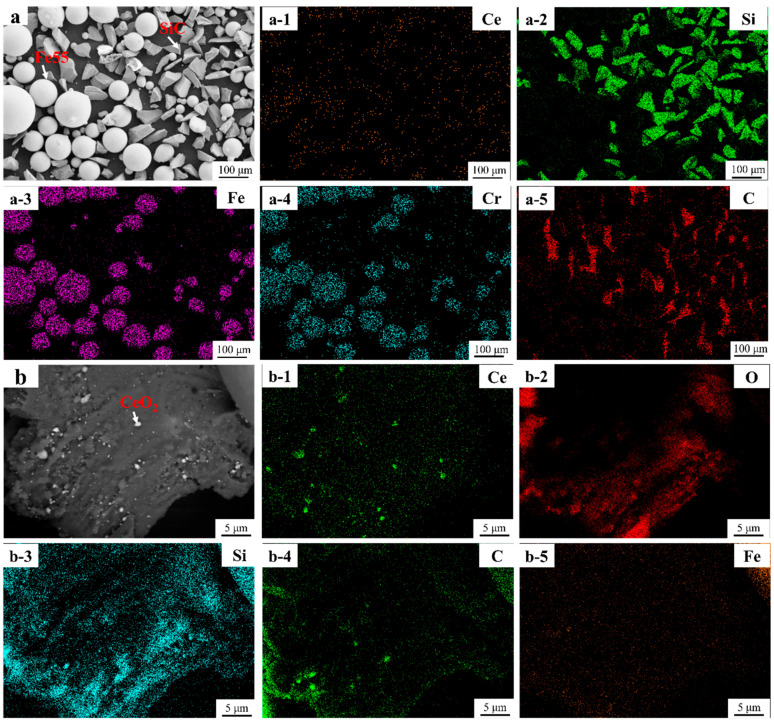
SEM micrographs and EDS mapping of Fe55+1.5 wt% SiC+0.01 wt% CeO_2_ (**a**–**a-5**) Fe55+1.5 wt% SiC+0.01 wt% CeO_2_ SEM micrographs and EDS mapping; (**b**–**b-5**) SEM micrographs and EDS mapping of SiC particle.

**Figure 5:**
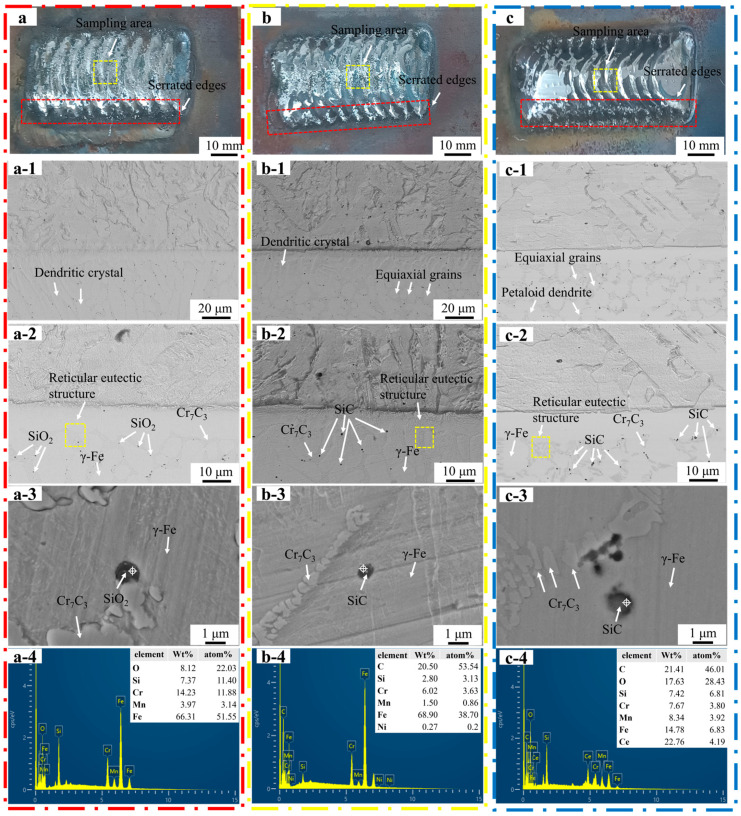
Macrograph and micrograph and EDS analysis of the welding layer. (**a**) Macrograph shape and sampling area of Fe55; (**b**) macrograph and sampling area of Fe55+1.5 wt% SiC; (**c**) macrograph and sampling area of Fe55+1.5 wt% SiC+0.01 wt% CeO_2_; (**a-1**,**a-2**) Fe55; (**b-1**,**b-2**) Fe55+1.5 wt% SiC; (**c-1**,**c-2**) Fe55+1.5 wt% SiC+0.01 wt% CeO_2_; (**a-3**,**a-4**) Fe55 EDS mapping; (**b-3**,**b-4**) Fe55+1.5 wt% SiC EDS mapping; (**c-3**,**c-4**) Fe55+1.5 wt% SiC+0.01 wt% CeO_2_ EDS mapping.

Figure 4 shows SEM micrographs and EDS mapping of Fe55+1.5 wt% SiC+0.01 wt% CeO_2_. Due to the low incorporation of CeO_2_, Ce was not present in the Figure 4(a-1). The Si-rich region overlapped with C, as shown in Figure 4(a-2,a-5). However, the C was not significant. An EDS analysis indicated that the white bright spots scattered on the surface of SiC in Figure 4b were CeO_2_. The white bright spots overlapped with the region of the Ce signal in Figure 4(b-1). The CeO_2_ with a maximum size of 500 nm had high surface energy resulting in the agglomeration of some CeO_2_. Due to the oxidation on the surface of the SiC, the signal of C in SiC was attenuated, leading to the interference of O in CeO_2_, as shown in Figure 5(b-2) and Figure 4(b-3). The Si- and C-enriched areas indicated that SiC showed a lamellar structure in Figure 4(b-3,b-4).

### 3.2. Morphology of Welding Layer

Figure 5a shows that the welding path at the surface of Fe55 welding layer was not clear; the jagged edges (red dashed line) on both sides exhibited the same characteristics. This indicated that it was filled by molten steel. The microstructures of the Fe55 welding layer in Figure 5(a-1) and Figure 5(a-2) were taken from the yellow dashed line in Figure 5a. The welding layer grain boundary was mainly composed of a reticular eutectic structure. The microstructure of Fe55 at the interface was mainly dendritic with a difference in grain size. The γ-Fe dendrites grew directionally from 1025 steel substrate into the welding layer. During the solidification process, the substrate side absorbed a large amount of heat input from the plasma arc to maintain a high temperature. The other side of the welding layer formed air convection due to the shielding gas agitation and took away the surface heat and maintained a low temperature. The temperature gradient perpendicular to the 1025 steel substrate provided a driving force for dendrite to grow. Therefore, the dendrite exhibited a directional growth pattern perpendicular to the substrate, which was proven in Liu et al.’s work [15]. The phase and EDS mapping of the Fe55 welding layer were shown in Figure 5(a-3,a-4); the Cr_7_C_3_ hard phases were small and formed a reticular eutectic organization with γ-Fe, since the welding layer was cooled fast. Although the Ar atmosphere isolated the oxygen, the [Si] reacted with [O] according to the reaction Equation (1) in the molten steel to inhibit the oxidation of [Fe] and avoid the generation of (FeO) inclusions according to the reaction Equation (2).

[Si] + [O] → (SiO_2_) (1)

[Fe] + [O] → (FeO)(2)

The SiO_2_ particles distributed along the grain boundaries had a size of 1~2 μm and agglomerated in some areas. The low melting point of SiO_2_ particles failed to inhibit grain growth.

Figure 5b showed the macrograph of Fe55+1.5 wt% SiC. Since the molten steel flowability decreased with SiC addition [10], the welding path and jagged edges (red dashed line) were clearer compared with Fe55. The sample was taken from the yellow dashed line in Figure 5b. The microstructures of the welding layer are shown in Figure 5(b-1,b-2). The grain boundaries consisted mainly of a reticular eutectic structure [16]. The grains were composed of γ-Fe [16]. The reticular eutectic structure was mainly composed of γ-Fe and Cr_7_C_3_ [13]. The microstructure was dendrites and equiaxed grains. The dendrites grew directionally with the temperature gradient as a driving force. In Figure 5(b-2), SiC particles were found on the grain boundaries and inside the grains. The number of dendrites was reduced, indicating that the growth of dendrite was inhibited by the particles and formed equiaxed grains. The SiC decomposed to [Si] and [C] at high temperatures according to the reaction Equation (3).

SiC → [Si] + [C](3)

The [Si] content in the molten steel rose and refined the grains [16]. Moreover, the EDS mapping results of Figure 5(b-3) show that the spherical particles were SiC. This indicated that the SiC were not completely melted and became nucleation points of γ-Fe. The SiC on the grain boundaries inhibited the grain boundary expansion. Therefore, the addition of SiC lead to the formation of equiaxed grains.

Figure 5c shows a macrograph of the welding layer of Fe55+1.5 wt% SiC+0.01 wt% CeO_2_. The welding path and serrated edges were clearest in all samples. The sample was taken from the yellow dashed line in Figure 5c. The microstructures are shown in Figure 5(c-1,c-2). The grain boundaries were mainly composed of a eutectic structure. CeO_2_ increased the viscosity of the molten steel. The SiC was difficult to agglomerate after stirring and dispersion by the Ar airflow. The distribution of SiC was uniform and they acted as nucleation points of γ-Fe, promoting the formation of equiaxed grains. When the number of grains reached the limit of surface energy, the equiaxed grains fused with each other to form petaloid grains in order to reduce the surface energy [17]. In Figure 5(c-1), SiC particles were uniformly distributed inside the welding layer and observed on the grain boundaries and inside the grains. Since the formation of rare earth compounds containing Ce and O was through nano CeO_2_ [18], the heterogenous nucleation of primary carbides was promoted. This resulted in a larger size of Cr_7_C_3_ hard phases than other samples. Some SiC agglomerated, as shown in Figure 5(c-2). Although the co-addition of CeO_2_ and SiC increased the viscosity of the molten steel, the collisions and agglomeration of SiC were inevitable under heating and stirring conditions in the molten steel. Figure 5(c-3,c-4) show the phase and EDS mapping results of the Fe55+1.5 wt% SiC+0.01 wt% CeO_2_ welding layer. The Ce-enriched areas were observed on the SiC particles inside the grains, where a eutectic structure of Cr_7_C_3_ and γ-Fe was formed, as shown in Figure 5(c-3). However, the high [O] content was due to the high activity of CeO_2_ adsorbed in the enrichment of inclusions.

**Figure 6:**
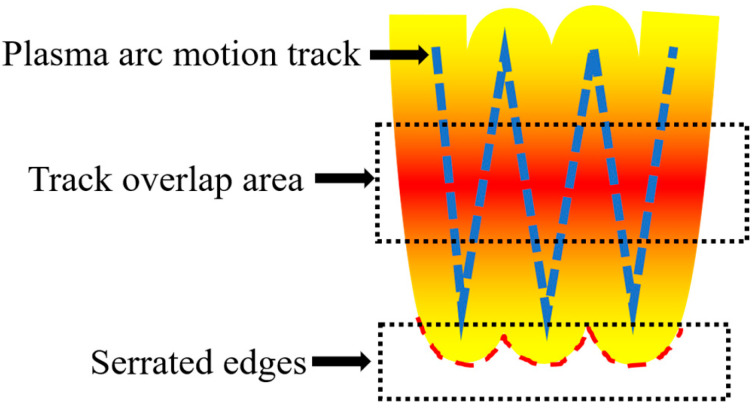
Schematic diagram of welding layer.

A schematic diagram of the welding layer is shown in Figure 6. The sides of the welding layer formed a jagged edge (red dashed line). The plasma arc paths (blue dashed line) partially overlapped and formed an overlapping molten pool. The overlapping molten pools had a higher temperature gradient [19]. The molten steel in these areas where the paths overlapped expanded to each other and were influenced by the viscosity of the molten steel. The molten steel with low viscosity expanded easily and the welding paths and jagged edges were not clearly visible. The molten steel with high viscosity was difficult to expand. Therefore, the welding paths and jagged edges were clearly visible.

**Figure 7:**
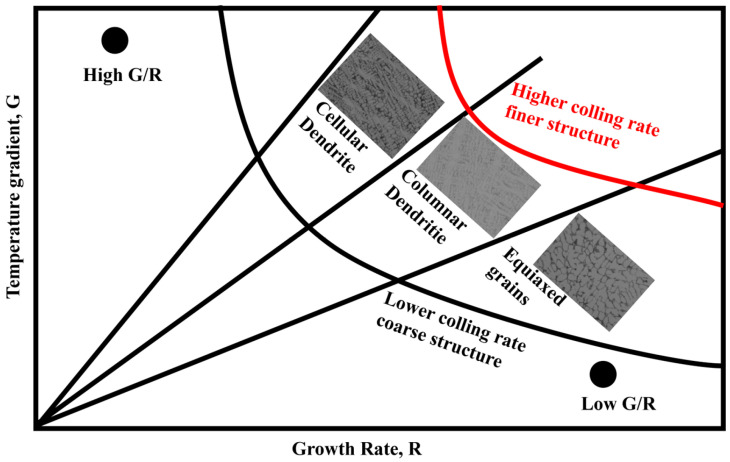
Effect of G and R on the morphology and dimensions of the solidified microstructure.

The changes in the solidification microstructure can be explained by Figure 7 and Equation (4), where the liquid temperature gradient was *G_L_*, the solidification rate was *R*, and the undercooling Δ*T_S_* was assumed to be constant, due to the high temperature of the plasma arc at 3000 °C. The addition of SiC or CeO_2_ affected the flowability of the molten steel at a macro level. This led to a reduction in the liquid phase diffusion coefficient *D_L_* of Cr and C, which resulted in a decrease in the *G/R* ratio, as shown in Figure 7. The Fe55 had a higher *G/R* ratio and no SiC or CeO_2_ to inhibit the dendritic growth. This resulted in the microstructure mainly being composed of dendrites. The diffusion coefficient *D_L_* of Cr and C elements in molten Fe55, Fe55+1.5 %wt SiC, and Fe55+1.5 wt% SiC+0.01 wt% CeO_2_ decreased with an increase in SiC or CeO_2_. Since Cr and C atoms were components of the eutectic structure, the decrease in the diffusion coefficient led to a uniform distribution of Cr and C atoms. This provided a rapid replenishment of atoms for grain boundary formation during the equiaxed crystallization process, which explains the differences in microstructures observed in different samples. Moreover, the drag effect on grain boundaries caused by Ce addition also inhibited the formation of dendrite grain boundaries [10]. This effect also explained the flowability decrease in the molten steel at a macro level.

(4) $$\frac{G_{L}}{R}>\frac{∆T_{s}}{D_{L}}$$

**Figure 8:**
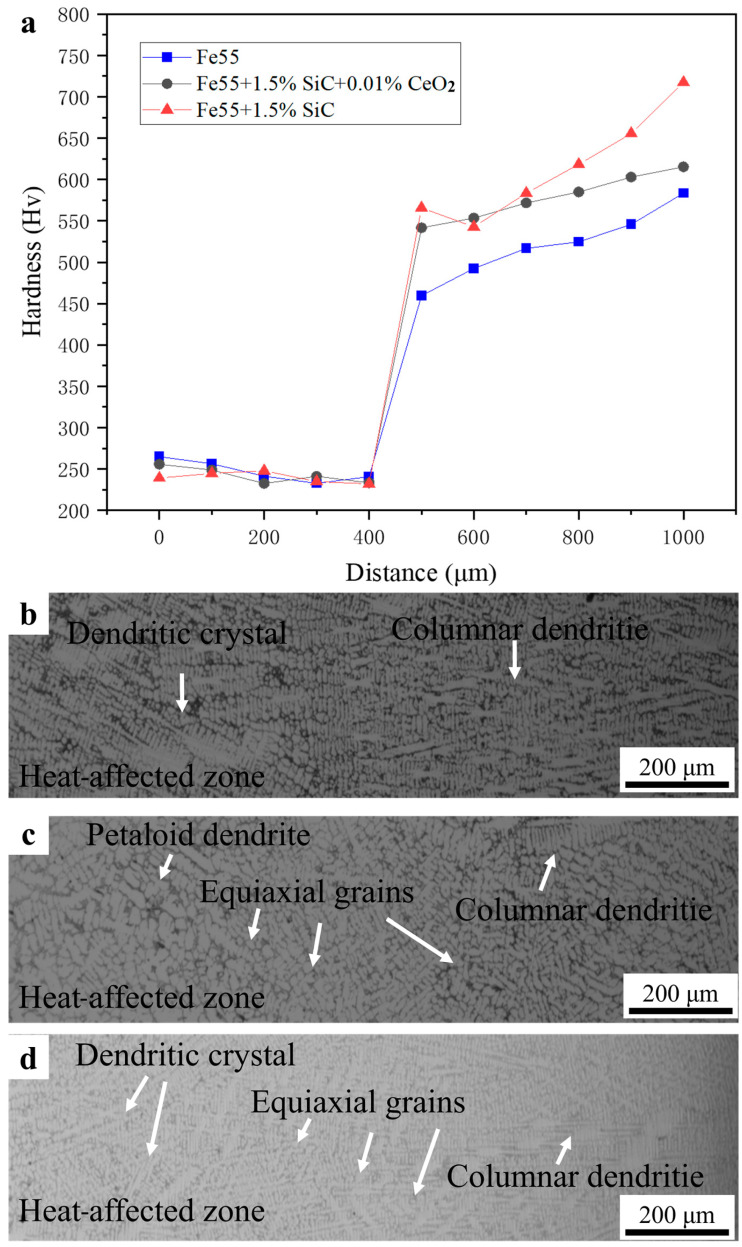
Microhardness test and welding layer interface. (**a**) Microhardness; (**b**) Fe55; (**c**) Fe55+1.5 wt% SiC+0.01 wt% CeO_2_; (**d**) Fe55+1.5 wt% SiC.

The γ-Fe was mainly strengthened by (Cr,Fe)_7_C_3_ carbides. The SiC in the welding layer and the formation of Cr_7_C_3_ could improve the hardness of the welding layer. Due to the relatively low density of some (Cr,Fe)_7_C_3_ near the fusion line in Fe55, there was a fluctuation in microhardness in Figure 8a. The coarse grains can be observed in the heat-affected zone in Figure 8b and the microstructures were mainly composed of dendrites. In Fe55+1.5 wt% SiC, although the non-uniform distribution of SiC particles resulted in uneven distribution of (Cr,Fe)_7_C_3_, the grains of γ-Fe were refined with the increasing content of nucleation sites and [Si, C] in Figure 8d. The addition of CeO_2_ caused the enhance phase to be uniformly distributed in γ-Fe, which lead to the transitional states of the microstructure in Figure 8c.

### 3.3. Wear Morphology

**Figure 9:**
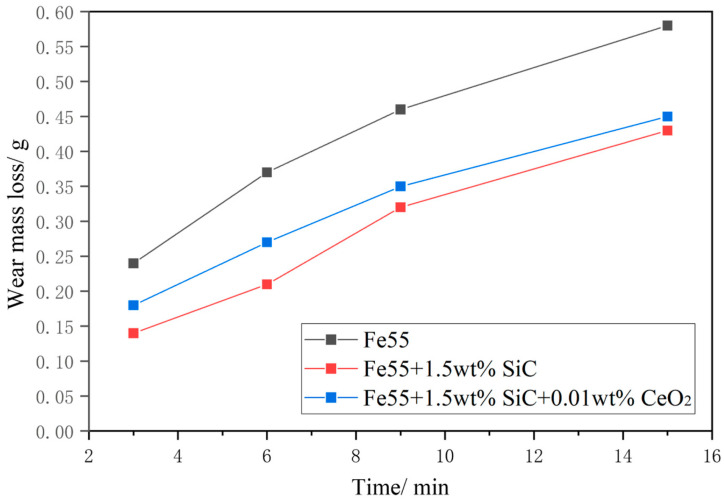
The wear mass loss of the sample after a 15 min test under 20 N.

Figure 9 shows the test results of the wear mass loss of the sample after a 15 min test under a load of 20 N. The changes in wear mass loss exhibited a similar trend, with the slope initially increasing and then decreasing as the wear time was prolonged. The wear mass loss of Fe55 was significantly higher than that of other samples. The sample with SiC addition exhibited the lowest mass loss. This was because SiC floated to the surface of the welding layer during the spraying process and aggregated, resulting in an increase in the surface hardness and enhancement in the wear resistance. As the wear progressed, the contact area increased, leading to a decrease in the wear rate at 9 min. However, at a wear time of 6 min, there was a slight increase in the wear rate. The wear mass loss of the Fe55+1.5 wt% SiC+0.01 wt% CeO_2_ sample remained stable, but due to its lower surface hardness compared to the sample with only SiC added, the wear mass loss of this sample was higher.

**Figure 10:**
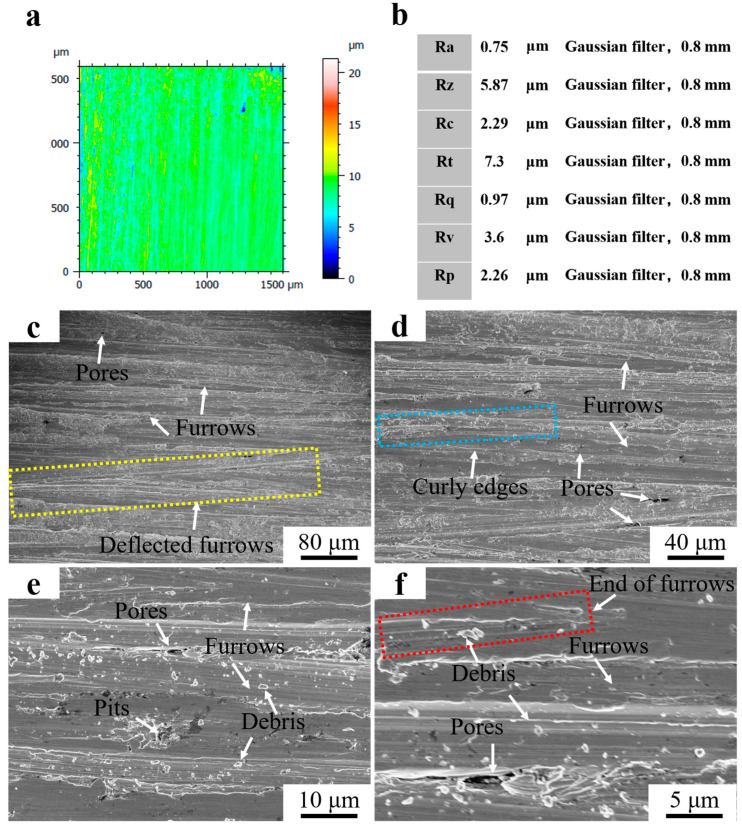
Roughness and morphology of Fe55 worn surface. (**a**) 3D morphology of the worn surface; (**b**) Surface roughness value; (**c**) ×250; (**d**) ×500; (**e**) ×2000; (**f**) ×5000.

Figure 10a shows the 3D morphology of the surface of Fe55 after a wet friction test. The depth of the furrows on the friction surface was marked by color with a non-uniform distribution. The blue area represents deep furrows as well as holes. The yellow and red areas represent light furrows. The structure of the blue areas was mainly γ-Fe with a low density of Cr_7_C_3_ enhancement, which wore easily. Defects such as inclusions or porosity existed in these areas, which resulted in the fatigue wear and the formation of spalling pits. The Cr_7_C_3_ agglomerated in the red region and increased the hardness. Therefore, the furrows were shallow. According to the ISO 21920-2:2021 standard [20], the Ra was 0.75 μm and Rz was 5.87 μm at a Gaussian filter of 0.8 mm. The abrasion roughness was between N5 and N6, as shown in Figure 10b. Figure 10c–f shows the worn surface micrograph of Fe55 after the wet friction test. Furrows and holes can be observed in Figure 10c. However, the furrows were deflected (yellow dashed line), which was a result of the movement of abrasive SiC being blocked by the Cr_7_C_3_ hard phase. In terms of abrasive wear, the hardness of the abrasive SiC was higher than γ-Fe. Then, the abrasive SiC was deflected toward the γ-Fe region with a low density of Cr_7_C_3_. The edges of the furrows were clearly curled and demonstrated a cut characteristic (blue dashed line). This was formed by the abrasive SiC during the cutting of the low hardness γ-Fe. In Figure 10e, the hidden porosities caused by the casting defects were exposed during the friction process. The fatigue wear on the friction surface resulted in spalling pits. In addition, there was a large amount of abrasive debris on the surface, which were hard three-bodied embedded in soft γ-Fe during movement and could not be washed away. Levy at el examined how, when the particles are strong enough not to break up on impacting, the erosion rate became constant [21]. This explained the reason why abrasive SiC embedded in soft γ-Fe. The size of the abrasive debris was significantly different. Part of the abrasive debris was newly generated, which indicated that not only abrasive wear but also three-body wear had occurred during wet friction [22]. The end point of partial furrows was visible (the red dashed line) in Figure 10f, which was the result of the abrasive SiC being blocked by the Cr_7_C_3_ hard phase and being unable to deflect to the nearby area.

**Figure 11:**
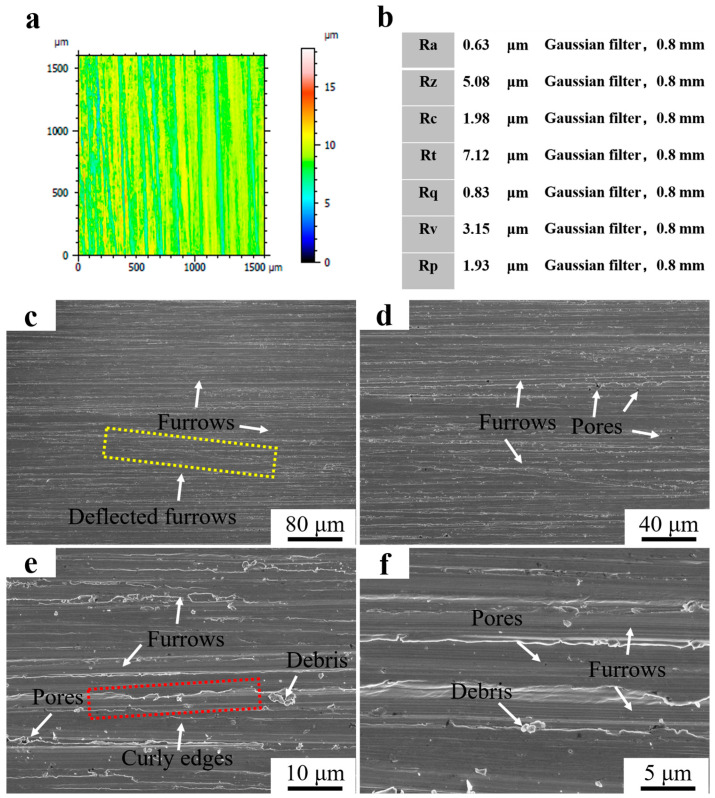
Roughness and morphology of Fe55+1.5 wt% SiC worn surface. (**a**) 3D morphology of the worn surface; (**b**) surface roughness value; (**c**) ×250; (**d**) ×500; (**e**) ×2000; (**f**) ×5000.

Figure 11a shows a 3D micrograph of Fe55+1.5 wt% SiC after the wet friction test. The color distribution of the worn surface was more uniform after the addition of SiC. The blue area represented the deep furrows, and the microstructure was mainly γ-Fe with a low density of Cr_7_C_3_. The yellow and red areas represent the lighter furrows with Cr_7_C_3_ and SiC. The SiC floated up to the surface and agglomerated. The [C] generated by the decomposition of SiC promoted the growth of Cr_7_C_3_ and increased the surface hardness. According to the ISO 21920-2:2021 standard, the Ra was 0.63 μm and Rz was 5.08 μm at a Gaussian filter of 0.8 mm. Therefore, the roughness was between N5 and N6, as shown in Figure 11b. Figure 11c–f shows the surface micrograph of Fe55+1.5 wt% SiC after the wet friction test. Dense furrows can be observed in Figure 11c, indicating that uniform wear occurred on the worn surface. However, there were still deflected furrows on the sample surface. The abrasive SiC was blocked by the Cr_7_C_3_ or SiC in γ-Fe and deflected toward the low hardness region. In Figure 11d, porosities can be observed, as Ar gas agitation led to the agglomeration of bubbles on the molten steel surface. Partially agglomerated SiC prevented the bubbles from breaking and led to casting defects. In Figure 11e, the significant curly edges on furrows are consistent with the cause of curly edges formation in the Fe55 sample. The number of debris was significantly reduced compared to the sample without the addition of SiC in Figure 10e. The addition of SiC increased surface hardness; this resulted in the abrasive not being able to embed in the γ-Fe and reduced the occurrence of three-body wear. Babu et al. indicated that the erosion mechanism changes depending upon the erodent type [23]. As the SiC addition increased the surface hardness, the three-body wear was reduced. The uniform size of the debris is visible in Figure 11f, indicating that the addition of SiC contributed to the wear uniformity.

**Figure 12:**
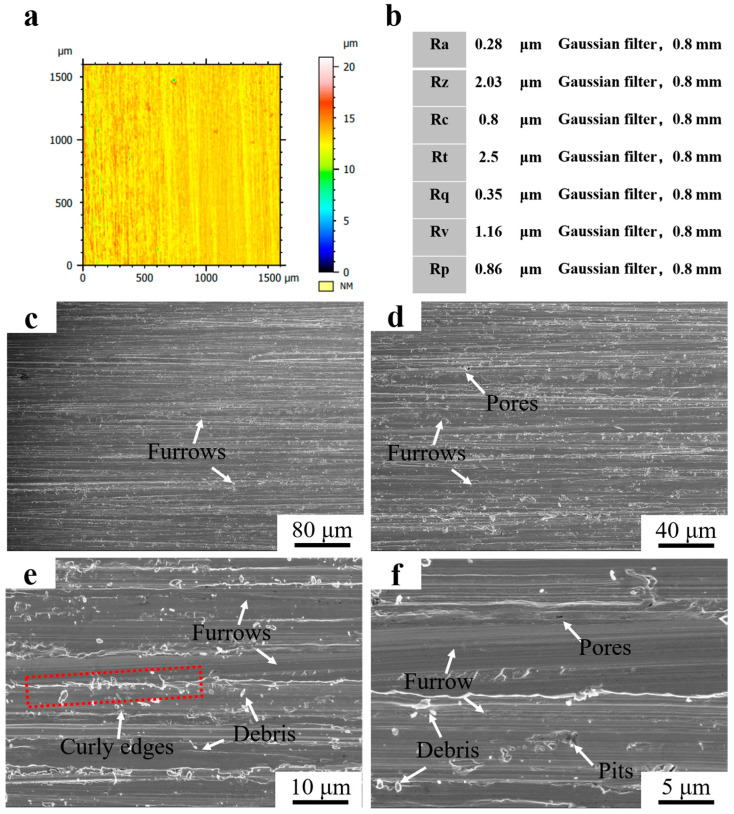
Roughness and morphology of Fe55+1.5 wt% SiC+0.01 wt% CeO_2_ worn surface. (**a**) 3D morphology of the worn surface; (**b**) Surface roughness value; (**c**) ×250; (**d**) ×500; (**e**) ×2000; (**f**) ×5000.

Figure 12a shows the 3D morphology of the surface after the friction test of Fe55+1.5 wt% SiC+0.01 wt% CeO_2_. The depth of farrows on the wear surface was marked by color. The color distribution of the wear surface was the most uniform among all the samples. The hardness of the red area was higher, where SiC or Cr_7_C_3_ showed aggregation. There were no deep farrows on the surface of this sample. CeO_2_ addition promoted the diffuse distribution of SiC and Cr_7_C_3_, which bore the friction load uniformly. According to the ISO 21920-2:2021 standard, the Ra was 0.28 μm and Rz was 2.03 μm at a Gaussian filter of 0.8 mm. The abrasion roughness was between N4 and N5, as shown in Figure 12b. Figure 12c–f shows the surface morphology of Fe55+1.5 wt% SiC+0.01 wt% CeO_2_ after the wet friction test. In Figure 12c, dense furrows on the surface indicate that this sample was worn uniformly. In Figure 12d, the number of holes is less than other samples in Figure 10d and Figure 11d. It was difficult for the stirring effect of Ar gas to form bubbles on the surface of the welding layer, since the viscosity of the molten steel increased by CeO_2_; this reduced the generation of defects. The furrows show significant curly edges in Figure 12e. A uniform size of the debris embedded in the surface was found. Therefore, the wear mechanism was abrasive wear and three-body wear [24]. The roughness and morphology results confirmed the research findings of Li et al. [13].

**Figure 13:**
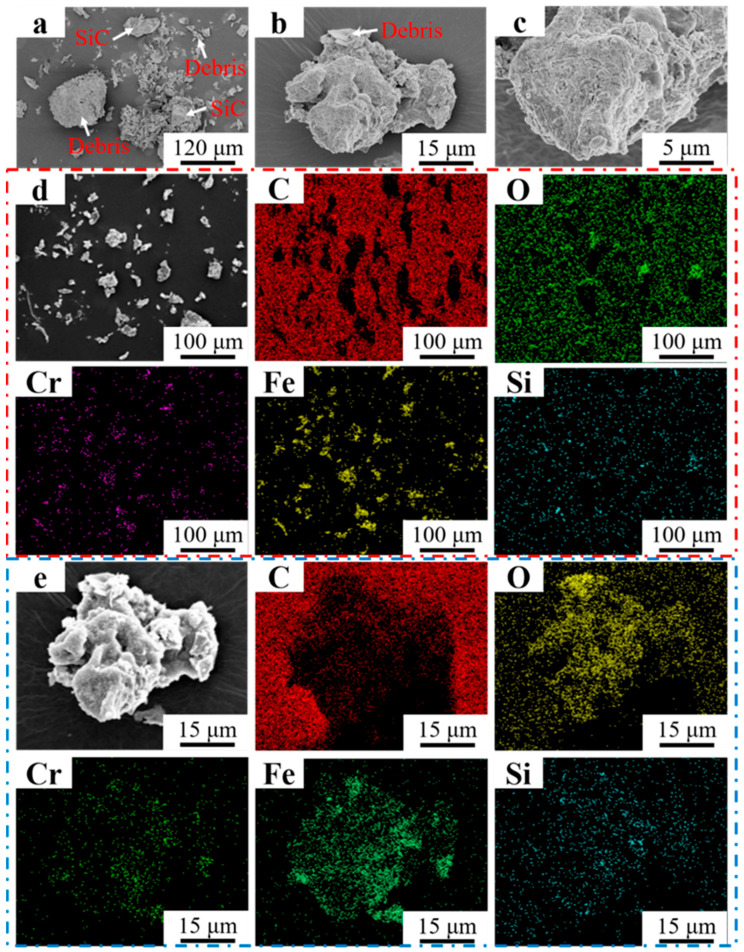
Morphology of Fe55 debris at different magnifications. (**a**) ×250; (**b**) ×2000; (**c**) ×5000; (**d**) EDS mapping ×250; (**e**) EDS mapping ×2000.

Figure 13a–c shows the morphology of the abrasive debris of Fe55 at different magnifications. The different sizes of the abrasive debris can be found in Figure 13a. Large debris was formed by the agglomeration of small debris. Meanwhile, the rest of the debris was of a lamellar structure, indicating that the abrasive debris was stripped from the worn surface by micro-cutting. Unbroken abrasive SiC could be observed. Large debris was formed by the lamellar debris and small abrasive debris in Figure 13b. The EDS mapping of Figure 13b is shown in Figure 13e. The results indicated that the main component of large debris was Fe_2_O_3_. The Fe reacted with O in the water to form fluffy Fe_2_O_3_ and adsorbed small debris to form big debris. In Figure 13d, the main components of debris were Fe and Cr. Due to the small size of the debris, it could be easily oxidized. Therefore, the O, Fe, and Cr elements’ enrichment areas overlapped. The SiO_2_ in the welding layer was stripped off, which resulted in Si enrichment being observed on the surface of the debris. The C and Cr enrichment areas overlapped in Figure 13e, indicating that the Cr_7_C_3_ was also worn and formed small debris, which explained the occurrence of three-body wear.

**Figure 14:**
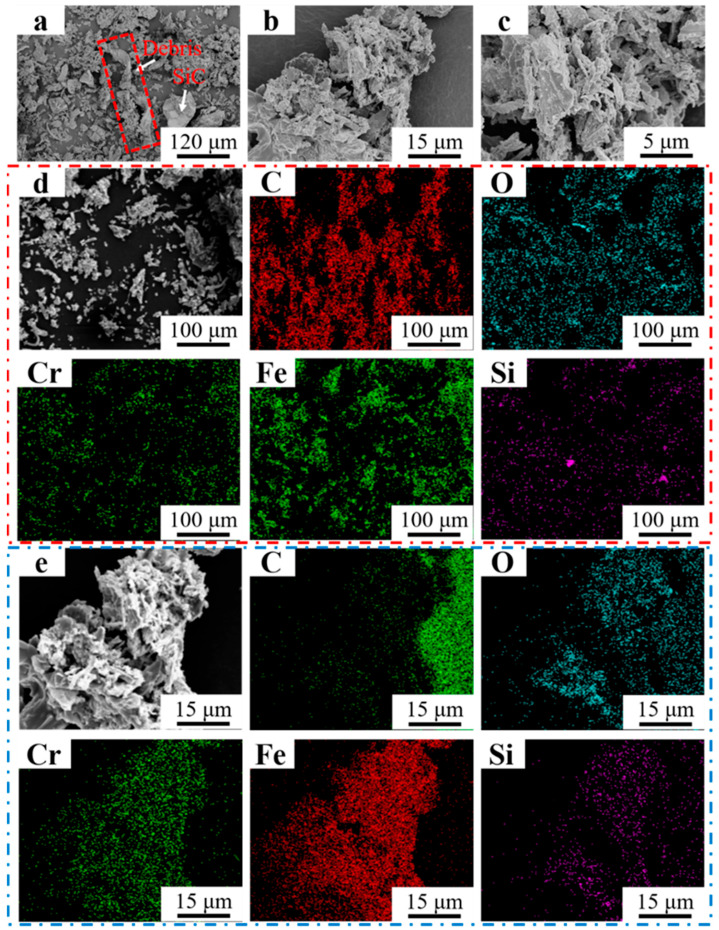
Morphology of Fe55+1.5 wt% SiC debris at different magnifications. (**a**) ×250; (**b**) ×2000; (**c**) ×5000; (**d**) EDS mapping ×250; (**e**) EDS mapping ×2000.

Figure 14a–c shows the morphology of the debris of Fe55+1.5 wt% SiC at different magnifications. Strip-shaped debris with lengths of 250~300 μm was marked by the red dashed line with a significant micro-cutting feature in Figure 14a, which suggested that abrasive wear was the main mechanism. SiC particles with sizes of 20~30 μm were also observed, which were produced from the collision and fragmentation between SiC abrasives and hard phases. Due to their small size and high surface energy, the agglomeration of the debris can be observed in Figure 14b,c. EDS mapping in Figure 14d indicates that the Si enrichment areas correspond to the fragmented SiC particles. Strip-shaped debris mainly consisted of Fe and Cr. The region enriched with Cr overlapped with C, indicating that the Cr_7_C_3_ was peeled off from the welding layer. Figure 14e shows the EDS mapping results of Figure 14b. Si and C enrichment were observed; this indicated that the SiC had not been fully decomposed and still bore the wear load. However, the increase in Si content reduced the toughness of the welding layer, which explained the reason for the strip-shaped debris cracking that can be observed in Figure 14a.

**Figure 15:**
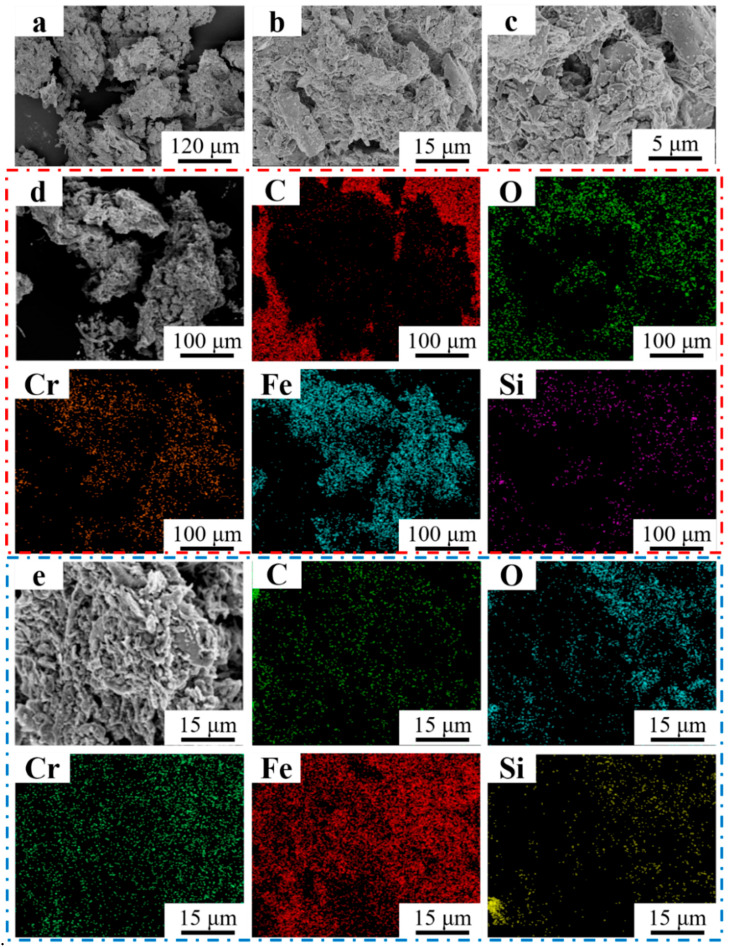
Morphology of Fe55+1.5 wt% SiC+0.01 wt% CeO_2_ debris at different magnifications. (**a**) ×250; (**b**) ×2000; (**c**) ×5000; (**d**) EDS mapping ×250; (**e**) EDS mapping ×2000.

Figure 15 shows the morphology of the debris of Fe55+1.5 wt% SiC+0.01 wt% CeO_2_ at different magnifications. Small debris with a flaky shape agglomerated to larger debris and represented micro-cutting characteristics, as shown in Figure 15a. The flaky shape debris, with a uniform size of 0.5~3 μm, as shown in Figure 15b,c, was cut uniformly by SiC abrasives. In Figure 15d, the flaky shape debris are mainly composed of Cr, Fe, Si, and O elements. However, Ce elements were not detected due to its low content. As the microstructure was significantly improved, the sample with the co-addition of CeO_2_ and SiC particles showed potential to manufacture uniformly worn sealing surfaces.

### 3.4. Wear Mechanism

The analysis of the SEM microstructure results in Figure 10, Figure 11, Figure 12, Figure 13, Figure 14 and Figure 15 indicate that the wear mechanism of the friction system was micro-cutting and micro-ploughing. During micro-cutting, SiC abrasives firstly formed furrows on the contact surface, causing hard Cr_7_C_3_ and soft γ-Fe to peel off from the contact surface. The amount of debris increased with test time. Although water served as the boundary lubricant for the contact surface, the debris inevitably embedded into the contact surface or escaped from the real contact areas via the gaps provided by the topographies of the worn surfaces within the contact zone [25]. This proved that all samples experienced three-body wear.

It is worth noting that, due to the high toughness of γ-Fe, micro-ploughing could dissipate frictional energy into microplastic deformation. Since water served as the boundary lubricant for the contact surface, fewer sharp abrasives were not enough to incur micro-cutting on the contact surface. Instead, they lost their kinetic energy through micro-ploughing, which dissipated the frictional energy into microplastic deformation and caused curly edges on furrows, as shown in Figure 10d, Figure 11e and Figure 12e. The region where micro-ploughing occurred underwent microplastic deformation in γ-Fe, but excessive microplastic deformation was inhibited by Cr_7_C_3_. Therefore, these γ-Fe did not peel off from the contact surface. However, the literature suggests that microplastic deformation caused by micro-ploughing leads to plastic fatigue wear [26], resulting in spalling pits caused by plastic fatigue wear in all samples.

When wear began, surface hardness played a key role, and higher hardness indicated stronger wear resistance. However, the increase in wear time resulted in microplastic deformation. When the hardness of the contact surface was excessively high and the toughness was too low, micro-cracks would form and propagate. Costin et al. indicated that the hardness and toughness of wear-resistant materials should be matched [27].

**Figure 16:**
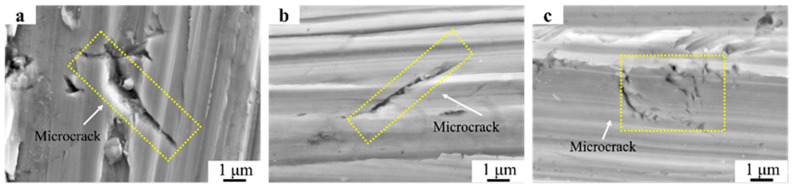
Microcracks after wet friction test. (**a**) Fe55; (**b**) Fe55+1.5 wt% SiC; (**c**) Fe55+1.5 wt% SiC+0.01 wt% CeO_2_.

Due to the predominance of dendritic microstructures and coarse grain size in the Fe55 welding layer, its hardness and toughness were the lowest among all the samples. Moreover, the middle region of the columnar grains was mainly γ-Fe. In the γ-Fe-enriched region, a larger micro-cutting amount occurred. These areas were located at the center of columnar grains, where no Cr_7_C_3_ hard phase existed internally. Therefore, the depth of γ-Fe corresponded to the length of the columnar grains. Once microcracks formed, their propagation could not be inhibited. Figure 16a shows the generation of the microcrack after the friction test on Fe55, with a length of 10 μm, and no evidence of microcrack termination was observed. This indicated that the Cr_7_C_3_ did not inhibit the microplastic deformation occurring in γ-Fe. The microcracks expanded with increasing wear time and eventually led to spalling, which explains why the wear mass loss of Fe55 was the highest among all the samples.

When SiC particles were added to the welding layer, due to its lower density compared to the molten steel, SiC floated to the surface of the molten steel during the melting process and provided nucleation points; this resulted in grain refinement. Although the microstructure of Fe55+1.5SiC was mainly dendritic, the refinement of the grains improved the toughness and increased the quantity of grains. The number of grain boundaries increased with the quantity of grains increased, which resulted in a more dispersed distribution of Cr_7_C_3_. Thus, the micro-ploughing occurring in the γ-Fe-enriched region might inhibited. SiC decomposed into Si and C. This increased the number of interstitial solute atoms in the surface of the welding layer and enhanced the hardness of γ-Fe. The high hardness prevented the embedding of abrasive particles but could potentially lead to a decrease in toughness. Since the hardness determined the wear mass loss in the early stage of friction, the Fe55+1.5 wt% SiC sample with the highest surface hardness exhibited the lowest wear loss initially. With the increase in wear time, plastic fatigue wear occurred in the γ-Fe-enriched region and γ-Fe started to peel off. Then, the hard Cr_7_C_3_ phase, losing support from the γ-Fe, would peel off. This explains the increase in wear rate in 6 min.

However, the results in Figure 5(b-2) indicate that the distribution of SiC was non-uniform. This resulted in regions with SiC agglomeration having higher microhardness, while areas without SiC had lower microhardness. The uneven hardness distribution made it easier for microcracks to expand in the SiC agglomeration regions, with a length of 7 μm, as shown in Figure 16b. However, the regions without SiC experienced deep furrows. Therefore, when the friction time reached 15 min, the wear mass loss of the Fe55+SiC sample was already approaching the Fe55+1.5 wt% SiC+0.01 wt% CeO_2_ sample in terms of lower surface hardness.

After the co-addition of SiC and CeO_2_, CeO_2_ decomposed into Ce and O during the spraying process. Ce hindered the agglomeration of SiC particles and the diffusion of C and Cr atoms. This resulted in the uniform distribution of C and Si in the molten pool. As a result, the microstructure and hardness uniformity were improved, thereby enhancing the resistance of micro-cutting and plastic fatigue wear. During micro-cutting, the uniformly distributed Cr_7_C_3_ hard phase prevented the excessive cutting of γ-Fe by abrasive particles. This reduced the micro-cutting volume and prevented the generation of more abrasive particles. In Figure 12f, there is no significant embedding of abrasive particles, as can be observed in Figure 10f. Figure 8c demonstrates that the microstructure and hardness of the welding layer only varied with temperature gradients. After the high-hardness region on the surface was worn, other regions could still maintain their hardness and toughness and avoided excessive wear losses. While micro-ploughing occurred, the increased toughness reduced the fatigue wear caused by microplastic deformation and the uniformly distributed Cr_7_C_3_ hard phase prevented excessive microplastic deformation. The microcrack shown in Figure 16c has a length of 3 μm and exhibits a distinct characteristic of being inhibited.

The CeO_2_ showed capabilities in grain refinement and in the promotion of equiaxed grain growth. However, the CeO_2_ content might depend on the types of enhancements, metal matrix, and application fields. Thus, the content of CeO_2_ should be well-matched with all the above factors. In this research, the addition of CeO_2_ sacrificed high hardness in exchange for a uniform microstructure; this might increase the wear mass loss in other application fields. In future works, the relationship of these factors should be further investigated to achieve better performances in specific fields.

## 4. Conclusions

(1) A co-addition of 0.01 wt% CeO_2_ and 1.5 wt% SiC increased the viscosity and decreased the G/R ratio of the molten steel. The distribution of SiC was uniform and they acted as nucleation points of γ-Fe, which promoted the formation of equiaxed grains.
(2) A co-addition of 0.01 wt% CeO_2_ and 1.5 wt% SiC reduced the liquid phase diffusion coefficient *D_L_*, resulting in nucleation points being uniformly distributed in γ-Fe. The temperature gradient perpendicular to the steel substrate provided a driving force for dendrite solidification; the dendrite exhibited a directional growth pattern perpendicular to the substrate, leading to the transitional states of microstructure.
(3) A co-addition of 0.01 wt% CeO_2_ and 1.5 wt% SiC promoted the diffuse distribution of SiC and Cr_7_C_3_, which bore the friction load uniformly. The SiC in the welding layer and the formation of Cr_7_C_3_ could improve the hardness of the surfacing layer. The grain boundaries consisted mainly of a reticular eutectic structure. The dense furrows on the surface indicated that the samples were worn uniformly. The welding layer of Fe55+1.5 wt% SiC+0.01 wt% CeO_2_ could effectively improve the service life and long-term sealing performance of the valve spools.
(4) The sub-wear mechanism of the friction system was micro-cutting and micro-ploughing and the uniform distribution of Cr_7_C_3_ could prevent excessive microplastic deformation, indicating that the hardness and toughness of Fe55+1.5 wt% SiC+0.01 wt% CeO_2_ was well-matched.

## Data Availability

Data are contained within the article.
